# National Association of Dental Faculties

**Published:** 1888-10

**Authors:** 


### National Association of Dental Faculties, 1888, Louisville, Kentucky.

The National Association of Dental Faculties convened in fifth annual session Monday, August 27, 1888.

The following colleges were represented:

Baltimore College of Dental Surgery, B. Holly Smith, W. W. Foster.

Boston Dental College, J. A. Follet.

Chicago College of Dental Surgery, Geo. H. Cushing, A W. Harlan, Truman W. Brophy, J. N. Crouse.

Kansas City Dental College, J. D. Patterson.

Missouri Dental College, W. H. Eames.

New York College of Dentistry, Frank Abbott.

Ohio College of Dental Surgery, Grant Mollyneaux.

Harvard College, Dental Department, Thos. Fillebrown.

Pennsylvania College of Dental Surgery, C. N. Pierce.

Philadelphia Dental College, S. H. Guilford.

University of Iowa, Dental Department, A. O. Hunt.

University of Pennsylvania, Dental Department, James Truman, E. T. Darby.

Vanderbilt University, Dental Department, W. H. Morgan, H. W. Morgan.

Northwestern College of Dental Surgery, F. H. B. McDowell, E. J. Perry.

Louisville College of Dentistry, A. Wilkes Smith.

Northwestern University, Dental Dept., J. S. Marshall.

Indiana Dental College, J. E. Cravens.

Officers: President, A. O. Hunt; Vice-President, Thomas Fillebrown; Secretary, Junius E. Cravens; Treasurer, A. W. Harlan.

Executive Committee, Frank Abbott, J. Taft, S. H. Guilford.

Committee on Schools, Frank Abbott, S. H. Guilford, L. C. Ingersoll, R. B. Winder, Thomas Fillibrown.

The President, Dr. A. O. Hunt, occupied the chair. At this and subsequent sessions the principal subject agitated was the *extension of time* of college terms, and numerous resolutions were offered, discussed, amended, lost, laid on the table, laid over for one year, or carried.

On motion of Prof. C. N. Pierce, the roll of colleges was called for the purpose of ascertaining how many delegates had received instructions from their respective faculties as to the time they would agree to. The roll being called it was seen that delegates from eleven colleges came with definite instructions; nine were uninstructed.

On motion of Mr. McDowell, the roll was called of those who came with instructions as to the number of months, and number of terms favored by their respective colleges with the following result:

Harvard Univ., Den. Department, 3 yrs. of 9 mos. each.

Kansas City Den. College, 2 yrs. of 6 mos. each.

Missouri Den. College, 3 yrs. 7 or 9 mos.

Ohio College of Den. Surgery, 2 yrs. 6 mos.

Pennsylvania College of Den. Surgery, 2 yrs. 7 mos.

University of Michigan, Den. Department, 3 yrs. 9 mos.

University of Pennsylvania, Den. Department, 2 yrs. 7 mos.

Northwestern College of Den. Surgery, 3 yrs. 9 mos.

Louisville College of Dentistry, 3 yrs. 5 mos.

University of Tennessee, Den. Department, 2 yrs. 5 mos.

National University, Den. Department, 2 yrs. 6 mos.

In view of the fact that nearly one half of the delegates present had no instructions in the matter, definite action was postponed, and the following resolution, offered by Prof. Eames, and seconded by Prof. Cravens, was carried :

*Resolved:* That it is the sense of this meeting that the course of instruction in all colleges of this Association should be increased to three years of not less than five months each : and that delegates shall submit this question to their respective Faculties or College Boards, and reports of their action be made to this Association at its next annual meeting, in order that final action on this question may be had. *Adopted.*

Among the resolutions offered and seconded, and *held over for one year*, were the following, on the above subject:

From the Executive Committee Drs. Abbott, Taft, and Guilford :

Report—Whereas—It is the sense of this Association that less than two years of study and instruction, is insufficient to properly prepare any one for the practice of dentistry as demanded by the progress of the times; therefore;

*Resolved*, That at least two years of *bone fide* study, and attendance upon two full regular courses of instruction, in separate years, be required before graduation.

By Prof. Howe, Louisville College, seconded by Mr. McDowell:

*Resolved:* To substitute for resolution adopted at N. Y., 1884, the following—" Attendance upon three full regular courses of not less than five months each, in separate years, shall be required before examination for graduating; and no student shall be graduated until at least twenty-eight months after his first matriculation.

Proposed and seconded by the same;

*Resolved* to amend resolution adopted at New York, 1884, by inserting in the place of " one year's pupilage in a dental office" the words " one course of lectures in a dental college."

Offered by Prof. Howe and seconded by Prof. Taft;

*Resolved:* That recommendation New York, 1884: "That three years study of dentistry, including attendance upon two regular courses of lectures, be required previous to examination for graduation" be made mandatory.

Offered by Prof. J. S. Marshall;

*Resolved:* That no student shall be permitted to graduate until at least twenty-four months after matriculation.

Offered by Mr. F. N. B. McDowell and seconded by Prof. Pierce;

*Resolved:* That after the close of the scholastic year 1889–90 attendance upon three regular *winter* courses, of not less than six months each, held in separate years, be required of students by colleges in this Association, before examinations can be had for graduation.

The following resolution, offered by Professor Taft, and seconded by Prof. Brophy, was adopted;

*Resolved:* That hereafter a delegate representing a college of this Association shall be a member of the Teaching Faculty of such college; and shall present properly executed credentials specifying his authority to represent and act for such college before he shall be entitled to vote on questions before this Association.

Resolution offered by Professor Guilford (Phil. College) and adopted;

*Resolved:* That when from sickness or death in his family, a student has been prevented from taking his intermediate examination and of obtaining his certificate from the institution where his first course was attended; such student may be examined for entrance to the senior class of any college of this Association upon presentation of a certificate from the Dean or Secretary of the school at which said student attended such first course, assenting to such examination.

Prof. W. W. Foster (Balt. Col. Den. Surg.) presented a series of questions in regard to the privilege of a student who has failed in his junior examination, or who for any cause has failed to present himself for the same. Has he the privilege of a fall examination, previous to the opening of the senior classes, either before the Faculty of the college where he passed his junior year, or by the Faculty of another college? and does passing successfully such examination entitle him to enter the senior class? If he fails to pass such examination by the Faculty of one college can he go to another college and pass another examination, entering the senior class of the second college?

After some discussion of these questions, the communication was referred to a Committee consisting of Profs. Fillebrown, Patterson, and Smith, their report being substantially to the effect that any college Faculty might examine a junior student as often as they chose previous to the beginning of the next session, and that the student is entitled to as advanced a standing as his examination warrants.

Of the Committees on text books Prof. Fillebrown, Char. of Com. on Operative Dentistry reported progress, the work being in the hands of the printer; Prof. Guilford reported that the work on Orthodontia would probably be issued by the 1st of January, 1889.

Dr. W. X. Sudduth was authorized to prepare and publish a text book on Dental Embryology and Histology.

The following officers were elected:

President, A. O. Hunt, Iowa City, Dent. Dept. Univ., Iowa.

Vice-President, L. D. Carpenter, Atlanta, Ga., Den. Dept. Southern Med. Col.

Secretary, J. E. Cravens, Indianapolis, Ind., Dent. Col.

Treasurer, A. W. Harlan, Chicago, Chicago Dent Col.

Executive Committee, Frank Abbott, Chairman, New York City, N. Y. Dent. Col.

J. Taft, Cincinnati, Univ. Mich. Dent. Dept.

S. H. Guilford, Philadelphia, Phil. Den. Col.

Com. *ad interim*,

Thos. Fillebrown, Chrm. Boston, Harvard Univ. Dent. Dept.

I. T. Crawford, Nashville, Univ. Tenn. Dent. Dept.

T. W. Brophy, Chicago, Chicago Dent. Col.

The Committee on Schools was continued.

Adjourned to meet at time and place of American Dental Association, Saratoga, N. Y., Aug. 5, 1889, 9:30 a. m.

"MRS. M. W. J.'

